# Trimethoprim-Sulfamethoxazole Plus Azithromycin to Prevent Malaria and Sexually Transmitted Infections in Pregnant Women With HIV (PREMISE): A Randomized, Double-Masked, Placebo-Controlled, Phase IIB Clinical Trial

**DOI:** 10.1093/ofid/ofae274

**Published:** 2024-05-08

**Authors:** Jodie A Dionne, Judith Anchang-Kimbi, Jiaying Hao, Dustin Long, Tobias Apinjoh, Pius Tih, Rahel Mbah, Edward Ndze Ngah, Jonathan J Juliano, Mauricio Kahn, Katia Bruxvoort, Barbara Van Der Pol, Alan T N Tita, Jeanne Marrazzo, Eric Achidi

**Affiliations:** Department of Medicine, Division of Infectious Diseases, University of Alabama at Birmingham, Birmingham, Alabama, USA; Center for Women's Reproductive Health, University of Alabama at Birmingham, Birmingham, Alabama, USA; Department of Parasitology and Immunology, University of Buea, Buea, Cameroon; Departments of Biostatistics and Epidemiology, School of Public Health, University of Alabama at Birmingham, Birmingham, Alabama, USA; Departments of Biostatistics and Epidemiology, School of Public Health, University of Alabama at Birmingham, Birmingham, Alabama, USA; Department of Parasitology and Immunology, University of Buea, Buea, Cameroon; Cameroon Baptist Convention Health Services, Cameroon Health Initiative at UAB, Bamenda, Cameroon; Cameroon Baptist Convention Health Services, Cameroon Health Initiative at UAB, Bamenda, Cameroon; Cameroon Baptist Convention Health Services, Cameroon Health Initiative at UAB, Bamenda, Cameroon; Department of Medicine, Division of Infectious Diseases, University of North Carolina at Chapel Hill, Chapel Hill, North Carolina, USA; Department of Medicine, Division of Infectious Diseases, University of Alabama at Birmingham, Birmingham, Alabama, USA; Department of Medicine, Division of Infectious Diseases, University of Alabama at Birmingham, Birmingham, Alabama, USA; Departments of Biostatistics and Epidemiology, School of Public Health, University of Alabama at Birmingham, Birmingham, Alabama, USA; Department of Medicine, Division of Infectious Diseases, University of Alabama at Birmingham, Birmingham, Alabama, USA; Center for Women's Reproductive Health, University of Alabama at Birmingham, Birmingham, Alabama, USA; Department of Obstetrics and Gynecology, Division of Maternal-Fetal Medicine, University of Alabama at Birmingham, Birmingham, Alabama, USA; Department of Medicine, Division of Infectious Diseases, University of Alabama at Birmingham, Birmingham, Alabama, USA; Department of Parasitology and Immunology, University of Buea, Buea, Cameroon

**Keywords:** *Chlamydia trachomatis* (CT), intermittent preventive treatment for malaria in pregnancy (IPTp), *Neisseria gonorrhoeae* (NG), *P. falciparum*, syphilis

## Abstract

**Background:**

This trial tested the effectiveness of a novel regimen to prevent malaria and sexually transmitted infections (STIs) among pregnant women with HIV in Cameroon. Our hypothesis was that the addition of azithromycin (AZ) to standard daily trimethoprim-sulfamethoxazole (TMP-SMX) prophylaxis would reduce malaria and STI infection rates at delivery.

**Methods:**

Pregnant women with HIV at gestational age <28 weeks were randomized to adjunctive monthly oral AZ 1 g daily or placebo for 3 days and both groups received daily standard oral TMP-SMX through delivery. Primary outcomes were (1) positive peripheral malaria infection by microscopy or polymerase chain reaction and (2) composite bacterial genital STI (*Chlamydia trachomatis, Neisseria gonorrhoeae*, or syphilis) at delivery. Relative risk and 95% confidence intervals were estimated using 2 × 2 tables with significance as *P* < .05.

**Results:**

Pregnant women with HIV (n = 308) were enrolled between March 2018 and August 2020: 155 women were randomized to TMP-SMX-AZ and 153 women to TMP-SMX-placebo. Groups were similar at baseline and loss to follow up was 3.2%. There was no difference in the proportion with malaria (16.3% in TMP-SMX-AZ vs 13.2% in TMP-SMX; relative risk, 1.24 [95% confidence interval, .71-2.16]) or STI at delivery (4.2% in TMP-SMX-AZ vs 5.8% in TMP-SMX; relative risk, 0.72 [95% confidence interval, .26-2.03]). Adverse birth outcomes were not significantly different, albeit lower in the TMP-SMX-AZ arm (preterm delivery 6.7% vs 10.7% [*P* = .3]; low birthweight 3.4% vs 5.4% [*P* = .6]).

**Conclusions:**

The addition of monthly azithromycin to daily TMP-SMX prophylaxis in pregnant women living with HIV in Cameroon did not reduce the risk of malaria or bacterial STI at delivery.

## Teaser

In this trial, 308 pregnant women with HIV in Cameroon were randomized to standard daily trimethoprim-sulfamethoxazole ± monthly azithromycin. Primary outcomes defined as the proportion of women with malaria or bacterial sexually transmitted infections at delivery were similar in both groups.

## BACKGROUND

Malaria and bacterial sexually transmitted infections (STIs) are among the most prevalent infections in pregnancy in sub-Saharan Africa (SSA), particularly among women with HIV [1]. In 2021, 95% of global malaria cases occurred in SSA, including 13 million cases in pregnant women [2]. Malaria is endemic in Cameroon where transmission of the parasite *Plasmodium falciparum* occurs year round, and HIV prevalence in pregnancy averages 3.4% [3]. Despite increasing availability and use of insecticide-treated bednets (ITN), malaria prevalence during pregnancy and at delivery in Southwest Cameroon has been documented as 13% to 30% by peripheral blood microscopy or polymerase chain reaction (PCR) [4, 5]. In malaria-endemic areas, the World Health Organization recommends daily prophylaxis with trimethoprim-sulfamethoxazole (TMP-SMX 160 mg/800 mg; also known as cotrimoxazole) for pregnant women with HIV as intermittent preventive therapy during pregnancy (IPTp) to prevent malaria and opportunistic infections [6]. However, the efficacy of antifolate TMP-SMX malaria prophylaxis in pregnant women with HIV has been documented at 75% and the spread of parasites with antifolate drug resistance threatens efficacy further [7, 8].

It is a priority goal to identify new prophylactic regimens for malaria with safety and improved efficacy in pregnancy [9]. Azithromycin (AZ) is a well-tolerated antibiotic with a 68-hour half-life, activity against malaria, and a favorable safety profile in pregnancy [10]. AZ has been studied as an adjunctive IPTp agent in combination with chloroquine and dihydroartemisinin-piperaquine (DP) and it has activity against bacterial STI [11–13]. STI prevalence in pregnancy in SSA ranges from 3% to 37% for *Chlamydia trachomatis* (CT) and 1% to 21% for *Neisseria gonorrhoeae* (NG) [14]. Adverse birth outcomes of malaria and STI in pregnancy include low birthweight, preterm delivery, and neonatal infections but access to STI testing in SSA is limited [15–17]. Unlike IPTp, effective prophylaxis to prevent bacterial STI in pregnancy has yet to be identified and AZ has promise given its activity against CT, NG, and syphilis [18]. The PREMISE study (A Novel Regimen to PREvent Malaria and STI in Pregnant Women with HIV) was designed to compare the effectiveness and safety of adding monthly azithromycin to standard daily TMP-SMX in pregnant women with HIV in Cameroon to prevent malaria peripheral parasitemia and bacterial STI assessed at delivery.

## METHODS

### Study Design

The PREMISE study was designed as a randomized, double-masked, placebo-controlled phase IIB pragmatic clinical trial. It was conducted by the Cameroon Health Initiative team with the University of Alabama at Birmingham (UAB), a collaboration between Cameroon and UAB. Study oversight was provided by institutional review boards (IRBs) in Cameroon and at UAB and an independent data safety monitoring board. All participants provided signed informed consent in English or French.

### Study Participants

Study nurses enrolled pregnant women with HIV from antenatal care clinics (ANCs) in settings in 3 regions of urban (Douala, Yaounde) and suburban (Mutengene) Cameroon. Participants were eligible if they had confirmed HIV infection, were aged ≥16 years, were gestational age <28 weeks according to dating criteria using ultrasound or last menstrual period, had live singleton pregnancy, and plans to follow-up and deliver at the enrollment facility. Exclusion criteria included severe anemia (hemoglobin <6 g/dL), known allergy to or intolerance of TMP-SMX or AZ, known congenital anomaly (detected by ultrasound), hospitalization or signs of labor at the time of enrollment, and prior severe cardiac disease.

### Randomization and Masking

Randomization into 2 prophylaxis groups stratified by facility was performed by a computer-generated block randomization sequence with variable size prepared in advance by the statistician. Only the study statistician and the study pharmacist were aware of assigned prophylaxis groups. A central pharmacist prepared AZ capsules 500 mg (Pfizer, New York, USA) and identical placebo for all study visits in advance using the predetermined numbered sequence. Participants received initial doses of study medication (2 capsules of azithromycin 500 mg or matching placebo) by directly observed therapy and were sent home with 4 additional tablets to complete a 3-day regimen of AZ 1 g or placebo taken by mouth once per day. Masking of the patient and team was maintained during subsequent follow-up visits because all medications were prepared in advance. All team members responsible for laboratory testing and outcome assessment were masked to the randomization arm.

### Procedures

At the enrollment visit, women were randomized, completed a survey, and had blood collected for testing. Women were advised to take their antiretroviral therapy (ART) daily and TMP-SMX prophylaxis daily as prescribed and sufficient medication supply was ensured. Medication adherence for TMP-SMX/AZ and ART was assessed at each follow-up visit by self-report using the 4-question, validated medication adherence questionnaire, and pill-count [19]. ITN, condoms, and safe sex counseling were also offered at each visit. Appointments were given for monthly follow-up visits to collect additional medication and a phone card. Study visits were scheduled to coincide with ANC visits.

Participants with illness or symptoms were encouraged to present for an unscheduled sick call visit. Malaria infection diagnosed at baseline or during sick call visits was treated according to national guidelines with quinine in the first trimester and injectable artesunate in the 2nd and 3rd trimesters. STI infections diagnosed were treated according to Centers for Disease Control and Prevention treatment guidelines. At the near-term visits (35-42 weeks’ gestation) and delivery visit, blood was collected for malaria parasitemia and syphilis testing with a provider-collected vaginal swab for STI testing and anorectal swab for GBS (Group B streptococus) testing. Placental testing is not included because samples were only available for a small subset of participants. Women who delivered outside the facility were invited to return within 14 days for sample collection (with samples included in the at-delivery group). For participants who did not deliver at the facility and did not complete the postpartum visit, samples collected at the near-term visit were used for endpoint determination. Participants were contacted by phone within 6 weeks of delivery to assess for additional birth outcomes.

### Laboratory Procedures

Malaria testing was performed by blood smear microscopy at each study visit and rapid diagnostic testing (HRP-2 [Abbott Bioline, IL, USA] or pLDH-based test for *P falciparum* [Access Bio Inc., NJ, USA]) for sick call participants with fever or other symptoms of malaria, according to routine practice on site. Thick blood smears were prepared and stained with 10% Giemsa. Malaria parasitemia status (asexual or sexual stages) was determined under oil immersion with the 100× objective of a binocular microscope. Blood smears were considered negative if no parasites were found in 200 high-power fields. Microscopy was performed at the facility with additional review at the University of Buea (UB) Parasitology lab. In case of discrepancy, UB results were included.

Dried blood spots from peripheral blood were also collected on filter paper for PCR testing at UB and the University of North Carolina at Chapel Hill. DNA was extracted from dried blood spots using Chelex. Falciparum malaria was detected using an ultrasensitive PCR for the multicopy target VarATS as previously described. The assay was carried out using Roche Universal Probe MasterMix on a BioRad CFX384, and otherwise unchanged [20]. PCRs were done in duplicate. PCR included negative controls and a standard curve to determine parasitemia. The standard curve was created using mocked dried blood spots containing whole blood and cultured 3D7 parasites (MRA-154). Control spots underwent the same extraction as clinical samples.

Blood was also collected for a complete blood count and syphilis testing. Per clinic protocol, syphilis screening was performed with a chromatographic immunoassay rapid test (ACON, CA, USA). Positive screens had follow-up rapid plasma regain (RPR; Omega Diagnostics, UK) and confirmatory *Treponema pallidum* Hemagglutination Assay (TPHA; Omega Diagnostics, UK), as indicated. Incident syphilis was defined as a new positive treponemal antibody with positive RPR or a 4-fold increase in RPR titer compared to the baseline titer. Vaginal swabs were stored in transport media for local *Streptococcus agalactiae* (GBS) nucleic acid amplification test (NAAT; GenXpert) and shipment to the UAB STI Laboratory for CT/NG/*Trichomonas vaginalis* NAAT (BD Max).

### Outcomes and Ascertainment

The primary study outcomes were the proportion of women with peripheral malaria infection at/near delivery by PCR or microscopy and the proportion of women with STI at/near delivery (combined CT/NG/incident syphilis).

Secondary outcomes included: mean infant birthweight, self-reported medication adherence for AZ/placebo and TMP-SMX (confirmed by pill count and pharmacy records), and medication tolerability (as recorded by study nurse with directly observed therapy or self-reported symptoms associated with additional dosing at home). Elicited symptoms were categorized as mild, moderate, or severe and included headache, dizziness, nausea, vomiting, abdominal pain, diarrhea, rash, and heart palpitations. Other secondary outcomes were symptomatic malaria parasitemia during the study, anogenital colonization with GBS by NAAT testing at delivery, and proportion with a composite measure of adverse birth outcomes: preterm delivery (<37 weeks), fetal death, low birth weight (<2500 g), or early neonatal death within 7 days.

Outcome ascertainment was performed by blinded experienced laboratory staff for malaria and STI testing and by blinded experienced maternity providers at the study facilities where women sought care.

### Statistical Analysis

To test our hypothesis that monthly AZ would reduce malaria infection at delivery and based on published data from Cameroon, we assumed a 30% peripheral malaria prevalence in the standard of care arm and a 15% prevalence in the AZ arm [4, 5]. A sample size of 268 women (134 per arm) would provide 80% power with a 2-sided 0.05 significance level using chi-square. With an additional assumption of 15% loss to follow up (knowing that facility delivery rates in Cameroon are as low as 60%), our goal was to enroll 308 women. For the coprimary STI outcome, based on data from Cameroon and other SSA countries, we assumed 30% composite STI positivity in the TMP-SMX arm (CT 20%; NG 5%, syphilis 5%) and 15% in the TMP-SMX/AZ arm, for 80% power and 2-sided alpha = 0.05 [15, 21, 22].

Descriptive statistics were used to compare the participants by intervention arm and the primary analysis was by intention to treat for the primary outcomes of proportion of women with parasitemia or bacterial STI. Categorical outcomes were compared by chi-square with Fisher exact tests as needed and *t*-test and Wilcoxon rank-sum tests for continuous outcomes. Lost to follow up was defined as participants with no study visit at/after 35 weeks and no outcome ascertainment. The intention-to-treat group was defined as women who were randomized and received at least 1 dose of study medication. The prespecified per protocol definition was participants with at least 1 follow-up visit, self-reported excellent (>90%) medication adherence, and study samples collected at or near delivery. Relative risk (RR) and 95% confidence interval (CI) were estimated using 2 × 2 tables with the PROC FREQ procedure. All analyses were performed in SAS v 9.4. The study was registered online at clinicaltrials.gov (NCT03431168). The funder of the study had no role in study design, data collection, data analysis, data interpretation, or writing of the report.

## PATIENT CONSENT STATEMENT

All participants provided written informed content before study participation and the study was approved by the IRB in Cameroon (the Cameroon Baptist Convention Health Services IRB) and in the United States (the University of Alabama at Birmingham IRB).

## RESULTS

**Figure 1.**
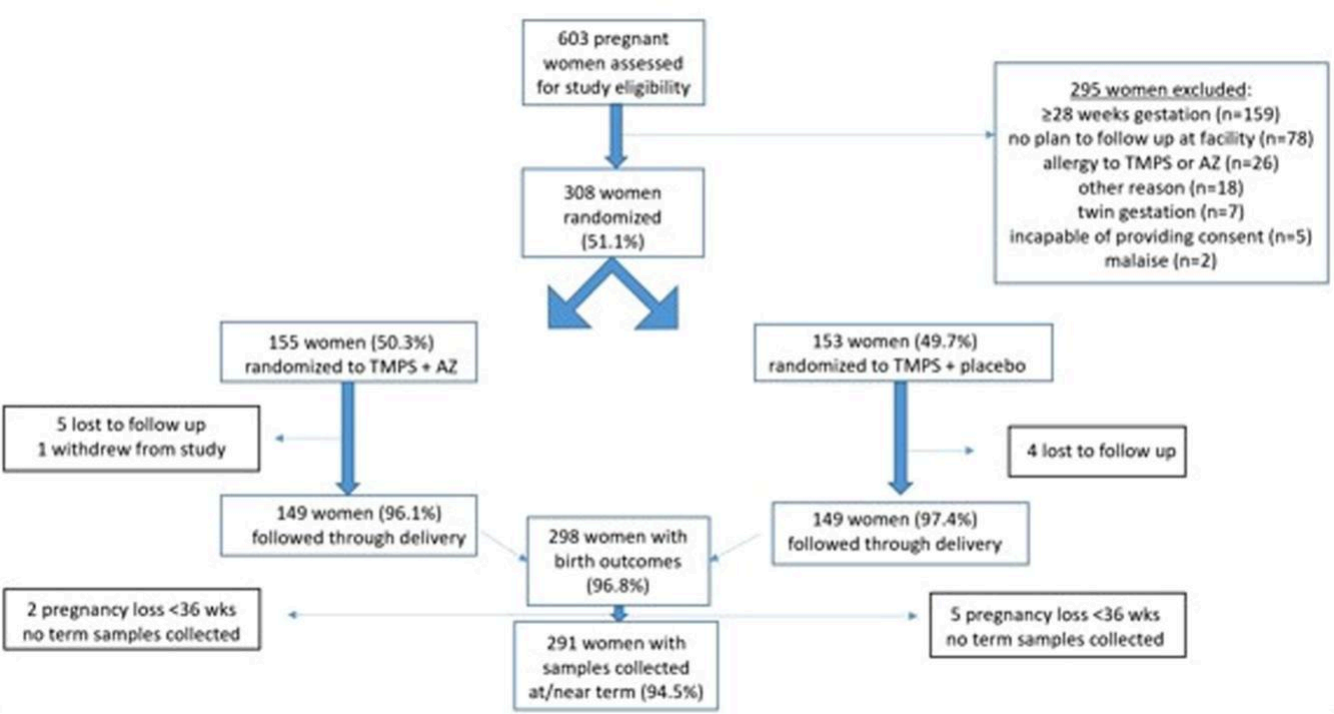
Flow diagram.

Among 603 pregnant women with HIV who were screened, 308 were enrolled at 3 facilities in Cameroon between 1 March 2018 and 30 August 2020 with follow up through January 2021 (Figure 1). In all, 155 women were randomized to the AZ/TMP-SMX intervention arm and 153 women were randomized to the placebo/TMP-SMX standard of care arm. A total of 298 women (96.8%) were followed through birth with similar loss to follow up in both groups. Because of early stillbirth or preterm delivery at home before 36 weeks’ gestational age (n = 2 in the TMP-SMX/AZ arm and n = 5 in the TMP-SMX arm), 291 women (94.5%; 291/308) had peripheral blood collected for malaria testing at delivery or near term.

Both groups were similar at baseline with median age 32 years, gestational age 20.8 weeks, 11.8% were primiparous, and 27.9% reported >5 pregnancies (Table 1). Most (71.5%) had been diagnosed with HIV >2 years earlier. All women (100%) reported taking ART, median CD4 count was 473 cells/mm^3^ (interquartile range 326-663) (n = 214) and HIV viral load was ≤200 copies/mL in 82.7% (129/156).

**Table 1.** Baseline Characteristics of Study Participants (n = 308)

Characteristic	Active (TMP-SMX—AZ) n = 155 n (%)	Placebo (TMP-SMX) n = 153 n (%)	*P* Value
Sociodemographics			
Median age in years (IQR)	32 (28-36)	32 (28-36)	.95
Mean BMI (SD)	27.8 (5.18)	28.6 (4.92)	.18
Enrollment site			.98
Douala (urban)	92 (59.4)	90 (58.8)	
Yaounde (urban)	18 (11.6)	18 (11.8)	
Mutengene (suburban)	45 (29.0)	45 (29.4)	
Highest level of education			.16
None/primary	48 (31.0)	41 (26.8)	
Secondary/high school	72 (46.5)	84 (54.9)	
University	35 (22.6)	28 (18.3)	
Employed in the past year	88 (56.8)	92 (60.1)	.63
Pregnancy history			
Mean gestational age in weeks (SD)	21.1 (5.0)	20.5 (5.5)	.29
Gestational age (completed weeks)			.12
<14	0 (0)	4 (2.6)	
14-20	48 (31.0)	48 (31.4)	
21-28	107 (69.0)	101 (66.0)	
Number of ANC visits to date*			.04
1	20 (13.0)	20 (13.2)	
2-3	125 (81.2)	110 (72.4)	
4+	9 (5.8)	22 (14.5)	
History of stillbirth	7 (4.5)	13 (8.6)	.23
History of preterm delivery	8 (5.2)	5 (3.3)	.60
HIV			
HIV diagnosis timing			.30
During this pregnancy	30 (19.4)	22 (14.4)	
<2 y ago, before pregnancy	21 (13.5)	15 (9.8)	
2-5 y ago	44 (28.4)	56 (36.6)	
>5 y ago	60 (38.7)	60 (39.2)	
HIV status of partner			.95
Positive	46 (29.7)	43 (28.1)	
Negative	81 (52.3)	82 (53.6)	
Unknown	28 (18.1)	28 (18.3)	
Current ART regimen^a^ (n = 247)			.52
NNRTI-based (efavirenz/NVP)	125 (98.4)	116 (96.7)	
Boosted PI-based (atz/r, drv/r, lop/r)	2 (1.6)	3 (2.5)	
INSTI-based (RTG, DTG)	0	1 (0.8)	
ART adherence past month by self-report			.03
Every day	150 (96.8)	139 (91.4)	
Most days	4 (2.6)	9 (5.9)	
Some days	1 (0.6)	4 (2.6)	
Malaria			
Bednet ownership	123 (79.4)	126 (82.4)	.50
Insecticide Treated Bednet (ITN)	110 (71.0)	116 (75.8)	.48
Bednet use on night prior	102 (65.8)	93 (60.8)	.36
Prescribed daily TMP-SMX	149 (96.1)	149 (97.4)	.76
Adherence to TMP-SMX (n = 297)			.05
Daily	133 (89.3)	114 (77.0)	
Often	6 (4.0)	12 (8.1)	
Sometimes	7 (4.7)	15 (10.1)	
Never	3 (2.0)	7 (4.7)	
Treated for malaria this pregnancy	16 (10.3)	19 (12.4)	.69
Peripheral malaria by microscopy	5 (3.2)	5 (3.3)	.98
Laboratory			
Median Hemoglobin (g/dL)	10.8 (9.9-11.5)	10.8 (10.1-11.6)	.24
Most recent CD4 count/mm³^b^ (IQR) (n = 214)	477 (308-664)	496 (360-655)	.65
Most recent HIV viral load* (n = 156)			.66
>200 copies/mL	16 (20.8)	11 (13.9)	
40–200 copies/mL	3 (3.9)	5 (6.3)	
<40 copies/mL	58 (75.3)	63 (79.7)	
Syphilis treponemal enzyme immunoassay positive	7 (4.5)	6 (3.9)	1.0

Abbreviations: ART, antiretroviral therapy; atv/r, atazanavir/ritonavir; drv/r, darunavir/ritonavir; DTG, dolutegravir; INSTI, integrase strand transfer inhibitor; IQR, interquartile range; lop/r, lopinavir/ritonavir; NNRTI, non-nucleoside reverse transcription inhibitor; NPV, nevirapine; PI, protease inhibitor; RTG, raltegravir; TMP-SMX, trimethoprim-sulfamethoxazole; SD, standard deviation.

^*^ *P* < .05.

^a^NRTI backbone: lamivudine or emtricitabine + tenofovir (n = 288); zidovudine (n = 12); unsure (n = 8).

^b^CD4 laboratory tests performed between January 2015 and December 2020. HIV viral loads dated from February 2017 to December 2019.

At baseline, 3.2% had malaria infection by microscopy, median hemoglobin was 10.8 g/dL (consistent with mild anemia), 73.4% had an ITN at home, 63.3% reported bed net use on the night prior, and 11.4% had been treated for malaria in pregnancy before enrollment. At baseline, 4.2% screened positive for syphilis by enzyme immunoassay and were treated and 98.7% reported 0 to 1 sex partners in the past 6 months.

At baseline, all women were prescribed ART. Reported daily ART adherence was 96.8% in the active arm versus 91.4% in the placebo arm (*P* = .03) and daily TMP-SMX adherence at baseline was 89.3% in the active arm versus 77.0% in the placebo arm (*P* = .046) (Table 1).

Of the 298 women (96.8% overall) with at least 1 follow-up visit during pregnancy, 84.1% had 2 visits and 49.0% had 3 visits with repeat dosing of monthly study medication. Reported daily adherence to TMP-SMX during the study was similar between both groups: 90.5% in the active group and 86.5% in the placebo group and 7.4% reported mild side effects with TMP-SMX. Nearly all women (99.0%) tolerated AZ well with directly observed therapy in clinic and 100% of women reported tolerating and completing the 3-day AZ or placebo regimen at home. Excellent daily ART adherence was reported by 95.9% (284/296) (Supplementary Table 1). Reported bed net use at follow-up visits was 69.4% and 3 women (all in the AZ arm) reported interim malaria diagnosed and treated between study visits. Sick call visits for acute symptoms were evenly distributed (11 per group): although fever (63.6% vs 36.4%), and malaria by RDT (rapid diagnostic test) (3/7 vs 0/5) were more common in the placebo arm compared to the active arm, differences were not significant. The 1 participant hospitalized for malaria was in the placebo arm.

Study outcomes are shown in Table 2 for the intention to treat analysis (n = 291). Peripheral malaria proportions by microscopy or PCR were 16.3% in the AZ/TMP-SMX arm and 13.2% in the placebo/TMP-SMX arm (RR, 1.24; 95% CI, .71-2.16) and composite STI proportions (CT/NG/syphilis) were 4.2% and 5.8%, respectively (RR, 0.72; 95% CI, .26-2.03). The per-protocol analysis was similar (n = 261) as well as a sensitivity analysis among women with parity 1 or 2. When stratified by parity, parasitemia was highest (averaging 19.1%) in women with 1 to 3 pregnancies and rates were similar in active and placebo arms. Among women treated for malaria in pregnancy before enrollment, 10% had peripheral parasitemia at or near delivery. For other infections detected with molecular diagnostics, the proportion with trichomoniasis were similar in both groups (2.4% in the TMP-SMX/AZ arm vs 2.4% in the TMP-SMX arm) as was anogenital colonization with *S agalactiae* (6.2% vs 5.7%). Other infections diagnosed at unscheduled sick call visits (pneumonia, urinary tract infection), were similar by arm (Table 3).

**Table 2.** Study Outcomes by Arm—Intention-to-Treat Analysis

Outcome	Active (TMP-SMX-AZ) N (%)	Placebo (TMP-SMX) N (%)	Relative Risk (95% CI)	*P* Value
Infection Outcomes at/near Delivery				
Peripheral Malaria by microscopy or PCR (N = 291)	N = 147	N = 144		
Composite PCR or Microscopy (N = 291)	24 (16.3)	19 (13.2)	1.24 (.71-2.16)	.51
PCR peripheral blood (N = 276)	9 (6.4)	7 (5.2)	1.25 (.48-3.26)	.80
Microscopy peripheral blood (N = 278)	16 (11.3)	13 (9.6)	1.18 (.59-2.36)	.70
Bacterial STI (N = 279)	N = 142	N = 137		
Composite STI (CT/NG/syphilis) (N = 279)	6 (4.2)	8 (5.8)	0.72 (.26-2.03)	.59
*Chlamydia trachomatis* (NAAT +) (N = 273)	2 (1.4)	2 (1.5)	0.92 (.13-6.46)	1.00
*Neisseria gonorrhoeae* (NAAT+) (N = 274)	1 (0.7)	0 (0)	NA	1.00
Incident syphilis (N = 278)	2 (1.4)	3 (2.2)	0.65 (.11-3.82)	.68
*Trichomonas vaginalis* NAAT + (N = 242)	3 (2.4)	3 (2.5)	0.95 (.20-4.62)	1.00
*Streptococcus agalactiae* anogenital NAAT + (N = 251)	8 (6.2)	7 (5.7)	1.08 (.40-2.89)	.88
Adverse pregnancy and neonatal outcomes	N = 149	N = 149		
Composite adverse birth outcome^a^	15 (10.1)	21 (14.1)	0.71 (.38-1.33)	.37
Miscarriage (<28 wk)	1 (0.7)	1(0.7)	1 (.06-15.84)	1.00
Stillbirth (≥28 wk)	1 (0.7)	3 (2.0)	0.33 (.04-3.17)	.62
Preterm delivery (<37 wk)	10 (6.7)	16 (10.7)	0.63 (.29-1.33)	.30
Low birthweight (<2500 g)	5 (3.4)	8 (5.4)	0.63 (.21-1.87)	.57
Neonatal death				
<7 d (early)	1 (0.7)	1(0.7)	1 (.06-15.84)	1.00
7-28 d (late)	0	2 (1.3)	NA	.50
Postpartum Maternal death	1 (0.7)	1 (0.7)	1 (.06-15.84)	1.00
Severe congenital anomaly	1 (0.7)	0	NA	1.00

Abbreviations: AZ, azithromycin; NA, not available; NAAT, nucleic acid amplification test; STI, sexually transmitted infection; TMP-SMX, trimethoprim sulfamethoxazole;

^a^Composite adverse birth outcome defined as miscarriage, stillbirth, preterm delivery, low birthweight, and/or early neonatal death.

**Table 3.** Events During Study Follow Up by Group

Interim or Visit Event	Active (TMP-SMX-AZ) (N = 391) n (%) or Median (Range)	Placebo (TMP-SMX) (N = 373) n (%) or Median (Range)	*P* Value
Routine follow up (N = 764)			
Number of follow-up visits	2 (1-5)	2 (1-6)	.39
Any bednet use last night	276 (71.5)	249 (67.3)	.21
Fever since last visit	14 (3.6)	13 (3.5)	.99
interim malaria diagnosis	3 (0.8)	0	.25
Took new medication	19 (4.9)	24 (6.4)	.48
Took ACT/quinine for malaria	4 (1.0)	5 (1.3)	.99
Source of malaria treatment			.06
Facility provider	8 (42.1)	10 (41.7)	
Outside provider	1 (5.3)	7 (29.1)	
Pharmacist	6 (31.6)	7 (29.1)	
Market	3 (15.8)	0	
Family member	1 (5.3)	0	
Hospitalization	2 (0.5)	0	.50
Unscheduled sick call visits (n = 22)	N = 11 (n %)	N = 11 (n %)	
Fever since last visit	4 (36.4)	7 (63.6)	.09
Symptoms (>1 allowed)			.99
Fatigue or myalgias	4 (36.4)	4 (36.4)	
Headache or dizziness	3 (27.3)	4 (36.4)	
Abnormal vaginal discharge	3 (27.3)	2 (18.2)	
Abdominal pain or diarrhea	2 (18.2)	2 (18.2)	
Cough	2 (18.2)	1 (9.1)	
Diagnosis at sick call visit			
Malaria	0	3 (27.3)	.21
UTI	2 (18.2)	2 (18.2)	.99
STI/vaginitis	2 (18.2)	3 (27.3)	.59
URI/pneumonia	2 (18.2)	1 (9.1)	.99
Positive malaria test (RDT)	0/5 (0)	3/7 (42.9)	.20
Hospitalized for malaria	0 (0)	1 (9.1)	.99

Abbreviations: ACT, artemisinin-based combination therapy; AZ, azithromycin; RDT, rapid diagnostic test; SMX, trimethoprim-sulfamethoxazole; STI, sexually transmitted infection; TMP-URI, upper respiratory infection; UTI, urinary tract infection.

In terms of birth outcomes (n = 298), rates of stillbirth (0.7% vs 2.0%; RR, 0.33; 95% CI, .04-3.17), preterm delivery (6.7% vs 10.7%; RR, 0.63; 95% CI, .29-1.33), and low birthweight (3.4% vs 5.4%; RR, 0.63; 95% CI, .21-1.87) were lower in the TMP-SMX/AZ arm versus TMP-SMX arm, respectively. The composite adverse birth outcome (miscarriage, stillbirth, preterm delivery, low birthweight, and/or early neonatal death) was 10.1% in the TMP-SMX/AZ arm compared to 14.1% in the TMP-SMX arm (RR, 0.71; 95% CI, .38-1.33; *P* = .37). There was 1 postpartum maternal death that occurred unexpectedly at home 6 days after delivery following traditional medicine for headache with no additional information available from the family. One infant born with severe urogenital congenital anomaly passed away in the hospital before 7 days of life.

## DISCUSSION

In this randomized placebo-controlled trial conducted among pregnant women with HIV in Cameroon, where malaria is holoendemic, the addition of monthly azithromycin before 28 weeks’ gestational age to standard daily TMP-SMX prophylaxis did not reduce malaria or bacterial STI infection rates at the time of delivery. TMP-SMX/AZ was well tolerated but infection rates were low and birth outcomes did not differ significantly between groups. An active clinical trial in Mali testing the role of azithromycin prophylaxis in preventing stillbirths and infant deaths will provide more information about AZ efficacy [23].

No other published study has focused on the combination of TMP-SMX and AZ prophylaxis in pregnant women with HIV. Our study findings are consistent with other randomized controlled trials (RCT) of novel IPTp malaria regimens among women with HIV in Africa. In Uganda, the addition of monthly DP starting in the 2nd trimester to daily TMP-SMX among women with HIV did not impact malaria prevalence, placental malaria, or birth outcomes compared to TMP-SMX alone (6.1% DP/TMP-SMX vs 3.1% TMP-SMX) although DP IPTp efficacy had been previously demonstrated in the same region among women without HIV [24, 25]. A recent RCT in East Africa (n = 4680) focused on preventing adverse birth outcomes in women without HIV did not show a benefit associated with DP IPTp (with or without AZ) compared to SP alone [13]. Other RCTs of IPTp with DP/TMP-SMX in women with HIV in Africa are under way [26]. Two older IPTp studies in women with HIV tested the addition of mefloquine to TMP-SMX with mixed results and poor mefloquine tolerability [27, 28]. An open-label RCT comparing AZ to SP starting at 16 weeks’ gestational age in Nigerian women with HIV found similar rates of peripheral parasitemia at delivery [29]. An older cross-sectional study in Malawi (n = 1121) in women with HIV showed odds ratio, 0.43 (95% CI, .19-.97) for malaria parasitemia with TMP-SMX compared to IPTp-SP [30]. Although emerging *P falciparum* SP antifolate resistance is of concern, the efficacy of first-line TMP-SMX prophylaxis in women with HIV persists [6, 31, 32].

Malaria rates in the PREMISE study were lower than anticipated. In addition to regional trends, ITN distribution, and access to free malaria treatment in pregnancy, rates in Cameroon may be partially explained by characteristics of the study population: women enrolled were mostly multiparous, urban residents with older age (>30 years), and higher socioeconomic status compared to other studies. Most participants were also well engaged in ANC care and HIV care with good virologic control, close follow up, and excellent reported medication adherence. In study design, we also may have overestimated the importance of HIV in terms of risk of malaria acquisition in pregnancy. In contrast to older literature where HIV was associated with 2- to 5-fold higher rates of malaria in pregnancy in SSA, current studies suggest that HIV may no longer be associated with increased risk [16, 33, 34]. This may reflect access to care and the success of HIV treatment programs in restoring host immunity. The epidemiology of malaria in SSA is also relevant to contextualize PREMISE study findings; a 30% to 40% reduction in incidence and prevalence has been documented between 2000 and 2015 [35]. These trends continued through 2019 and are thought to be multifactorial but attributed in large part to expanded access to and use of ITN in SSA. Consistent patterns have been noted in Southwest Cameroon where malaria parasitemia by microscopy among pregnant women without HIV decreased from 22% in 2013 to 13% in 2016 to 8% in 2023, whereas ITN usage increased from 17% to 68% [4, 36, 37].

Bacterial STI rates were also lower than anticipated. This may reflect characteristics of the study population. The association between CT, NG, syphilis in pregnancy, and adverse birth outcomes including low birthweight, preterm delivery, and congenital syphilis are clear yet the role of antimicrobial prophylaxis for STI to improve birth outcomes remains elusive. The prevention of adverse birth outcomes among women without HIV with AZ prophylaxis/treatment during pregnancy has had mixed impact in SSA. In Uganda, presumptive STI treatment with azithromycin, cefixime, and metronidazole reduced CT/GC by 57% and impacted preterm delivery rates but not birthweight [38]. In Malawi, adding AZ to IPTp-SP did not improve preterm delivery or birthweight compared to IPTp with SP alone [39]. In Papua New Guinea, SP-AZ was associated with improved birthweight compared to SP-CQ. One proposed mechanism was changes in inflammatory pathways and placental angiogenesis [40]. Another factor that could influence the impact of AZ prophylaxis for malaria and STI is resistance because of AZ mass drug administration used for trachoma control in endemic regions of SSA [41].

Because of lower than anticipated infection rates, an important study limitation was insufficient power to discern an association between the intervention and infection rates at delivery or birth outcomes. We also had a limited window for prevention because malaria and syphilis diagnosed at baseline were treated. Strengths include high rates of follow-up with no evidence of differential follow-up between the groups. Because differences between groups in terms of reported ART and TMP-SMX adherence were noted at baseline but not during follow-up, this was unlikely to differentially impact study findings. Subgroup analyses comparing participants with 1 follow up visit to >1 follow-up visit had similar study outcomes.

Future RCTs to test novel regimens to prevent malaria and STI in pregnancy are important and should include women living with and without HIV and target true high-risk groups, including focus on primigravidae. Selection of appropriate regimens to test requires careful consideration of risks and benefits and study design that includes microbiome sampling and antimicrobial resistance outcomes.

In conclusion, the addition of monthly azithromycin to daily TMP-SMX among pregnant women living with HIV in Cameroon in this RCT did not reduce the rate of malaria or bacterial STI at delivery. Medication was well tolerated and birth outcomes were not significantly different in both groups although limited by lack of power. Ongoing studies may provide additional insight about the impact of AZ use on birth outcomes in Africa.

## Supplementary Material

ofae274_Supplementary_Data

## Supplementary Data

Supplementary materials are available at *Open Forum Infectious Diseases* online. Consisting of data provided by the authors to benefit the reader, the posted materials are not copyedited and are the sole responsibility of the authors, so questions or comments should be addressed to the corresponding author.

## Notes

***Financial support*.** This work was supported by the Eunice Kennedy Shriver National Institute of Child Health and Human Development of the National Institutes of Health [1K23HD090993 to J.D.].
