# Supplementary material for: Trimethoprim-Sulfamethoxazole Plus Azithromycin to Prevent Malaria and Sexually Transmitted Infections in Pregnant Women With HIV (PREMISE): A Randomized, Double-Masked, Placebo-Controlled, Phase IIB Clinical Trial
Source: Open Forum Infect Dis. 2024 May 8;11(5):ofae274. doi: 10.1093/ofid/ofae274 (PMC11130525; doi:10.1093/ofid/ofae274)
Supplement: ofae274_Supplementary_Data [file ofae274_supplementary_data.docx]

**PREMISE SUPPLEMENTAL DATA**

**Supplemental Table 1 –Self-Reported Medication Adherence and Side Effects during study (n=296)**

| **Medication** | **Active**  **(N=148)**  **n (%)** | **Placebo**  **(N=148)**  **n (%)** | **p-value** |
| --- | --- | --- | --- |
| TMPS Adherence  Every day  Most days  A few days  Never | 133 (90.5)  12 (8.1)  1 (0.7)  1 (0.7) | 128 (86.5)  9 (6.1)  7 (4.7)  4 (2.7) | 0.08 |
| TMPS Side Effects | 13 (8.4) | 8 (5.9) | 0.50 |
| Tolerated AZ well in clinic | 146 (98.7) | 147 (99.3) | 1.00 |
| Completed AZ at home | 148 (100) | 148 (100) | 1.00 |
| Excellent Daily ART Adherence | 142 (96.2) | 142 (96.6) | 1.00 |
